# Monitoring progress towards the first UNAIDS target: understanding the impact of people living with HIV who re‐test during HIV‐testing campaigns in rural Mozambique

**DOI:** 10.1002/jia2.25095

**Published:** 2018-04-13

**Authors:** Laura Fuente‐Soro, Elisa Lopez‐Varela, Orvalho Augusto, Charfudin Sacoor, Ariel Nhacolo, Nely Honwana, Esmeralda Karajeanes, Paula Vaz, Denise Naniche

**Affiliations:** ^1^ Centro de Investigação em Saúde de Manhiça (CISM) Maputo Mozambique; ^2^ ISGlobal Barcelona Institute for Global Health Hospital Clínic ‐ Universitat de Barcelona Barcelona Spain; ^3^ Centre for Disease Control and Prevention (CDC) Maputo Mozambique; ^4^ Fundação Ariel Glaser Contra o SIDA Pediátrico Maputo Mozambique

**Keywords:** HIV care continuum, Linkage to care, Public health, Awareness, Disclosure, Sub‐Saharan Africa, Mozambique, 90‐90‐90 targets, HIV retesting

## Abstract

**Introduction:**

Awareness of HIV‐infection goes beyond diagnosis, and encompasses understanding, acceptance, disclosure and initiation of the HIV‐care. We aimed to characterize the HIV‐positive population that underwent repeat HIV‐testing without disclosing their serostatus and the impact on estimates of the first UNAIDS 90 target.

**Methods:**

This analysis was nested in a prospective cohort established in southern Mozambique which conducted three HIV‐testing modalities: voluntary counselling and testing (VCT), provider‐initiated counselling and testing (PICT) and home‐based testing (HBT). Participants were given the opportunity to self‐report their status to lay counsellors and HIV‐positive diagnoses were verified for previous enrolment in care. This study included 1955 individuals diagnosed with HIV through VCT/PICT and 11,746 participants of a HBT campaign. Those who did not report their serostatus prior to testing, and were found to have a previous HIV‐diagnosis, were defined as non‐disclosures. Venue‐stratified descriptive analyses were performed and factors associated with non‐disclosure were estimated through log‐binomial regression.

**Results:**

In the first round of 2500 adults randomized for HBT, 1725 were eligible for testing and 18.7% self‐reported their HIV‐positivity. Of those tested with a positive result, 38.9% were found to be non‐disclosures. Similar prevalence of non‐disclosures was found in clinical‐testing modalities, 29.4% (95% CI 26.7 to 32.3) for PICT strategy and 13.0% (95% CI 10.9 to 15.3) for VCT. Prior history of missed visits (adjusted prevalence ratio (APR) 4.2, 95% CI 2.6 to 6.8), younger age (APR 2.5, 95% CI 1.4 to 4.4) and no prior history of treatment ((APR) 1.4, 95% CI 1.0 to 2.1) were significantly associated with non‐disclosure as compared to patients who self‐reported. When considering non‐disclosures as people living with HIV (PLWHIV) aware of their HIV‐status, the proportion of PLWHIV aware increased from 78.3% (95% CI 74.2 to 81.6) to 86.8% (95% CI 83.4 to 89.6).

**Conclusion:**

More than one‐third of individuals testing HIV‐positive did not disclose their previous positive HIV‐diagnosis to counsellors. This proportion varied according to testing modality and age. In the absence of an efficient and non‐anonymous tracking system for HIV‐testers, repeat testing of non‐disclosures leads to wasted resources and may distort programmatic indicators. Developing interventions that ensure appropriate psychosocial support are needed to encourage this population to disclose their status and optimize scarce resources.

## 1 Introduction

In 2014, UNAIDS set the ambitious global strategy of reaching the 90‐90‐90 targets to end the HIV epidemic by 2020 1. This plan established that 90% of the people living with HIV (PLWHIV) will know their HIV‐status, 90% of those will be on antiretroviral therapy (ART), and 90% of those on ART will reach viral suppression. There has been a 29% decline in new HIV infections in adults from 2010 to 2016 in eastern and southern Africa, even though, this region remains the most severely affected, where almost 19.4 million people are currently infected with HIV 2, 3. Despite the progress, important challenges remain unresolved. Globally, in 2016, 30% of the 36.7 million PLWHIV did not know their HIV‐status and 47% did not receive ART 3.

Awareness of HIV infection is the first critical step in the continuum of HIV care. However, it goes far beyond HIV serological testing and includes an understanding of the implications, acceptance of the diagnosis, willingness to disclose their status to health providers, family members and close community 4, 5, 6 and enrol and start in HIV care and treatment. Little is known about the extent and causes of non‐disclosure in different epidemic settings although it has an important impact both at an individual and at a public health level.

Non‐disclosure may lead to repeated HIV‐testing and/or repeated drop out and re‐engagement in HIV care after a positive HIV‐diagnosis 7. Efforts to quantify and describe testing inefficiencies will contribute to optimize resources to reach the 90‐90‐90 objectives in high burden areas of sub‐Saharan Africa. Accurate indicators of HIV awareness, linkage and retention in care need to take into account individuals that are aware of their HIV‐positivity but do not disclose their serostatus to the health provider. This study sought to characterize PLWHIV who fail to disclose their serostatus during clinical and community HIV‐testing campaigns in a rural district of Southern Mozambique.

## 2 Methods

### 2.1 Study area and population

The study was performed in the Manhiça District, a semi‐rural area in Maputo province, southern Mozambique. Since 1996, the Manhiça Health Research Centre has run a continuous health and demographic surveillance system (HDSS) for vital events including births and deaths, and migrations 8 which in 2015, at the time of the study, covered a total population of nearly 174,000 individuals 9, 10. This is a high HIV burden setting, with an estimated community based prevalence of 39.7% among adults in 2012 10, 11. Voluntary counselling and testing (VCT) and provider initiated counselling and testing (PICT) were the most widespread HIV‐testing modalities in the district, while home‐based testing (HBT) was not routinely performed as a programmatic HIV‐testing strategy. HIV‐testing and care is offered free of charge and criteria to start ART treatment followed the WHO recommendations 12. At each health facility, PICT and VCT are offered and routine patient‐level HIV clinical data are recorded in an electronic Patient Tracking System (ePTS), which allows monitoring of the HIV population registered in the facility, the quality of care provided as well as the retention, treatment adherence and occurrences of opportunistic infections. HIV‐testing is anonymous in Mozambique, and the current ePTS registers only HIV‐positive patients after they have attended their first clinical visit (further study area details on Appendix S1).

### 2.2 Study procedures and definitions

The current analysis was nested in a larger prospective observational linkage cohort offering HIV‐testing to adults through VCT, PICT and HBT between May 2014 and June 2015 in the Manhiça HDSS 13 (Appendix S1). The cohort inclusion criteria were adults over 18 years willing to participate, resident in the Manhiça District Hospital (MDH) catchment area, and receiving a first HIV‐positive result. Individuals at VCT and PICT were screened for eligibility after receiving an HIV‐positive result whereas HBT, patients were screened prior to testing. Individuals were excluded if they were pregnant women or co‐infected with tuberculosis. HIV rapid testing was performed following WHO/UNAIDS and national guidelines 14.

In order to be enrolled in the linkage cohort, all HIV‐diagnoses performed via VCT, PICT and HBT were verified for the absence of a previous HIV clinical chart registered in the ePTS available for three of district health facilities. The analysis of disclosure included those patients identified as having an HIV‐diagnosis prior to the testing campaigns, either found in the ePTS or self‐reported.

One thousand nine hundred fifty‐five subjects testing HIV‐positive through VCT and PICT were invited to participate in order to reach the sample size target for the linkage cohort.

Based on the sample size calculations (Appendix S1) of the linkage cohort study, HBT was attempted to be offered to 12,500 adults randomly selected from the all HDSS adult enumeration, of which 10,897 were visited at home and offered HIV counselling and testing (HCT)The HBT campaign took place in four field‐rounds and individuals were invited to participate in the linkage cohort prior to HCT 13. During the recruitment process, patients were given the opportunity to disclose their HIV‐status prior to counselling and testing when the counsellor asked up to two times about HIV‐testing history. HIV‐testing was offered to all individuals who did not disclose an HIV‐positive status.

For the purposes of this study, the following definitions were used:

- **Newly HIV‐diagnosed:** individuals testing HIV‐positive during the campaign and not previously registered in the ePTS.
- **Known HIV‐positive** *:*
  - **Self‐reported HIV‐positive:** individuals disclosing previous HIV‐positive diagnosis to the counsellor at any time prior to HIV‐testing. Only applicable for the HBT population.
  - **Non‐disclosure of HIV‐positive status:** individuals accepting HIV counselling and testing without revealing a previous HIV‐diagnosis. Non‐disclosure participants were identified among VCT, PICT and HBT strategies.
- **Loss to follow‐up (LTFU):** was defined as having the last clinical visit performed more than 180 days before the study visit.

### 2.3 Data collection and data management

Specific questionnaires, including information on awareness of HIV‐status, history of previous HIV‐testing, time since last HIV‐test and socio‐demographic information were designed for the study. Data from both clinical venues, VCT and PICT, were collected in paper format and double‐entered using the OpenClinica platform 15. As participants who self‐reported a previous HIV‐positive diagnosis were not included in the linkage cohort, only age and sex were recorded for those subjects. Data from the HBT venue were directly collected in electronic format in Open Data Kit software 1.4 (ODK) 16 at the time of the visit and uploaded into a database in REDCap (Research Electronic Data Capture), 17.

As a pilot, prior to the first round of HBT (HBT‐R1) a standard deterministic and probabilistic record‐linkage 18 was conducted in order to match the HDSS individuals simple randomized for HBT and the Manhiça hospital ePTS records for those already enrolled in care.

In the consecutive three HBT rounds individuals identified by that system were not visited in order to improve the efficiency and yield of HBT strategy.

Ascertainment of *known HIV‐positive individuals* was conducted by confirmation of the physical hospital identification card and/or location of individual's name in one of the three main district ePTS databases.

### 2.4 Statistical analysis

We conducted a descriptive analysis stratified by HIV‐testing modality, assessing proportions by Pearson and Fisher's exact chi‐square tests. For HBT analysis, data from the first round, where adults had the opportunity to disclose their serostatus to a study counsellor, was used for description and prevalence whereas data from rounds 1 to 4 were used for risk‐factor analysis. To assess potential risk factors associated with non‐disclosure to the health personnel, we estimated prevalence‐ratios (PR) through use of log‐binomial regressions 19, 20. Both unadjusted and adjusted PR (APR) were computed, and the analysis was conducted among the HBT participants and then among those who were enrolled in clinical care at the MDH. Age category (18 to 24, 25 to 34, 35 to 44, >45), sex, being previously enrolled in care and LTFU were included in the model as covariates. Confidence intervals for the binomial proportions were calculated through the Clopper‐Pearson method. Statistical analyses were performed using Stata 14.1 21.

### 2.5 Ethics

This study was approved by the Mozambican National Bioethics Committee as well as the Institutional Review Boards at the Hospital Clinic of Barcelona (Spain), the Manhiça Health Research Centre. It was also reviewed according to Centres for Disease Control and Prevention (CDC) human research protection procedures and was determined to be research, however CDC was not engaged directly with research participants. The purpose of the study was explained to participants and written informed consent was obtained.

## 3 Results

### 3.1 Study profile and baseline characteristics

**Figure 1:**
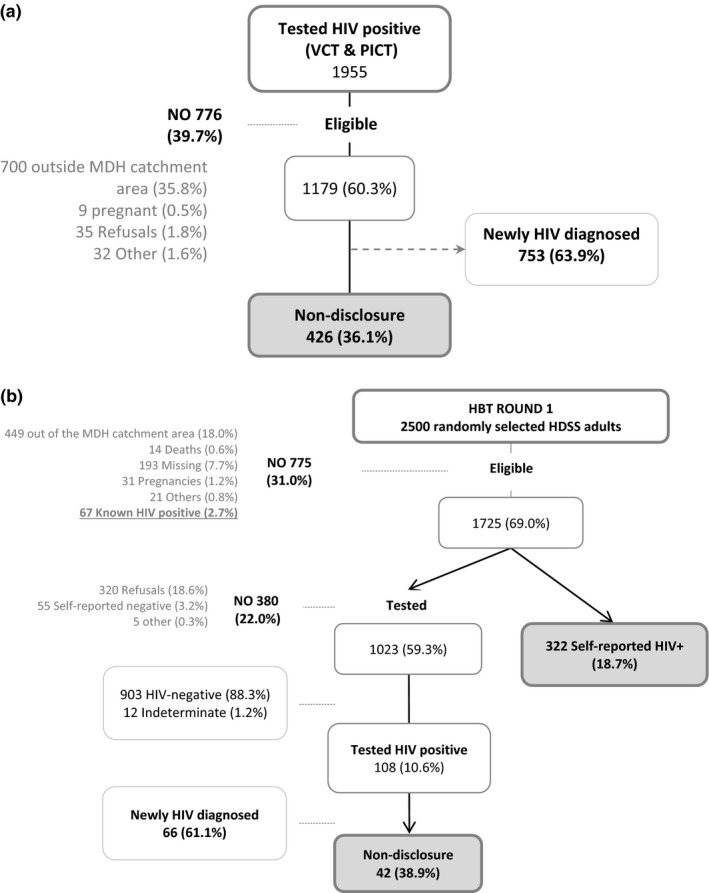
Study profile for (a) patients testing HIV‐positive in the clinic‐based testing strategies (VCT & PICT) and (b) patients testing HIV‐positive in the community strategy (HBT). Percentages are calculated over the previous step. Voluntary Counselling and Testing (VCT), Provider Initiated Counselling and Testing (PICT), Home‐Based Testing (HBT), Manhiça District Hospital (MDH) and Health Demographic Surveillance System (HDSS). HBT – Refers only to the first round of HBT (n = 2500).

During the hospital testing data collection phase, a total of 1955 people tested positive for HIV through VCT and PICT strategies. Of these, 39.7% (776/1955) did not meet the inclusion criteria for this analysis (Figure 1a). Of the remaining 1179 eligible individuals, 36.1% (426/1179) were later identified in the ePTS system as *known HIV‐positive* (118 individuals in VCT and 308 in PICT) and 63.9% (753/1179) were considered *newly HIV*‐*diagnosed* (Figure 1a).

From the individuals randomly selected and visited for HBT‐R1, 69.0% (1725/2500) were eligible for participation; 18.7% (322/1725) self‐reported their status, and were thus considered as *known HIV‐positive* individuals and not tested (Figure 1b). Of those 1023 undergoing HCT, 10.6% (108/1023) tested positive for HIV. After testing positive, 38.9% (42/108) of the individuals were identified by the ePTS system as *known HIV‐positive* (Figure 1b). The record‐linkage algorithm matched an ePTS record for 42.2% (182/431) of those defined as *known HIV‐positive* (either self‐reported or non‐disclosure) in HBT‐R1.

The median age of *known HIV‐positive* individuals from HBT, VCT and PICT was 37.3 years (IQR 29.0 to 44.0) and significantly higher (*p* < 0.001) for the HBT group 38.3 years (IQR 30.0 to 45.0) as compared to the PICT 32.5 years (IQR 26.0 to 37.0) and VCT 32.7 years (IQR 26.0 to 38.0). A significantly higher proportion of clients in PICT were men as compared to VCT and HBT (*p* < 0.001) (Table 1).

**Table 1:** Characteristics of *known HIV‐positive individuals* according to study HIV‐testing venue

		VCT		PICT		HBT‐R1		HBT‐All rounds		*p*
		118		308		431		2177		
		n	%	n	%	n	%	n	%	
Age category^a^	18 to 24	22	18.6%	58	18.8%	35	8.1%	214	9.8%	0.000
	25 to 34	56	47.5%	146	47.4%	141	32.7%	740	34.0%	
	35 to 44	28	23.7%	67	21.8%	137	31.8%	650	29.9%	
	45 to 54	6	5.1%	24	7.8%	78	18.1%	337	15.5%	
	55+	6	5.1%	12	3.9%	40	9.3%	236	10.8%	
Sex	Male	34	28.8%	132	42.9%	113	26.2%	566	26.0%	0.000
	Female	84	71.2%	176	57.1%	318	73.8%	1611	74.0%	
Serostatus	Non‐disclosed	118	100.0%	308	100.0%	42	9.7%	121	5.6%	0.000
	Self‐reported	NA	‐	NA	‐	322	74.7%	1207	55.4%	
	Determined by probabilistic record linkage (not visited)	NA	‐	NA	‐	67	15.5%	849	39.0	

VCT, Voluntary Counselling and Testing; PICT, Provider Initiated Counselling and Testing; HBT, Home‐Based Testing; NA, No Applicable.

a Age category in the PICT arm correspond to n = 307 participants.

### 3.2 Disclosure of HIV status to counsellors

From those participants included at HBT‐R1, a total of 431 were defined to be *known HIV‐positive individuals,* among which 74.7% (322/431) self‐reported their HIV‐status to the study staff and. Almost 10% (42/431) underwent HCT without disclosing their HIV‐positive status to the counsellor. An additional 15.5% (67/431) previously identified as HIV‐positive by the record‐linkage algorithm were not visited (Figure 1b).

Among those testing HIV‐positive in the VCT, PICT and HBT testing campaign, the proportion of non‐disclosures was highest in the HBT‐R1 arm, reaching 38.9% (95% CI 29.7 to 48.7), followed by the PICT at 29.4% (95% CI 26.7 to 32.3), and VCT at 13.0% (95% CI 10.9 to 15.3) (Table 2). VCT showed a significantly lower proportion of non‐disclosures as compared to the HBT and PICT (*p* < 0.001) (Table 2). The lower proportion in HBT‐all rounds as compared to HBT‐R1 is due to not visiting 39.0% (849/2177) of the individuals randomized to rounds 2 to 4 with a matched record in ePTS as mentioned in methods.

**Table 2:** Proportion of individuals across testing strategies (VCT, PICT and HBT) with an HIV‐positive result who did not disclose previous HIV‐positivity to counsellor

Testing venue	Total HIV‐positive tests	HIV Non‐disclosures	Non‐disclosures % (95% CI)	*p*
VCT	909	118	13.0% (10.9 to 15.3)	<0.0001
PICT	1046	308	29.4% (26.7 to 32.3)	
HBT‐R1	108	42	38.9% (29.7 to 48.7)	
HBT‐All rounds	490	121	24.7% (20.9 to 28.8)	

VCT, Voluntary Counselling and Testing; PICT, Provider Initiated Counselling and Testing; HBT, Home‐Based Testing.

### 3.3 Factors associated with non‐disclosure of HIV serostatus to the health personnel

We assessed factors associated with non‐disclosure among those who had the opportunity to disclose their serostatus over the four HBT rounds, thus excluding from the analysis those non‐disclosures in VCT and PICT. Of a total of 1328 *known HIV‐positive individuals*, 1207 were self‐reported and 121 were non‐disclosures (Figure S1). In a log‐binomial regression, younger age was significantly associated with non‐disclosure of HIV‐positive status (Table 3a). Individuals aged 18 to 24 years, had a 3.31 fold greater proportion (95% CI 1.91 to 5.74) of non‐disclosure as compared to the reference category of individuals over 45 years of age (Table 3a). The association was maintained for the 25 to 34 year‐old group (adjusted prevalence ratio APR 1.77, 95% CI 1.10 to 2.87).

**Table 3:** Factors associated with prevalence of non‐disclosure of HIV status in the HBT population in (a) the entire HBT population (n = 1328) or (b) those who were enrolled in clinical care at the MDH (n = 933)

		Prevalence						
		% (n/N)	PR	95% CI	*p*	APR	95% CI	*p*
a)								
Age category	18 to 24	20.41 (20/98)	3.69	2.11 to 6.45	<0.001	3.31	1.91 to 5.74	<0.001
	25 to 34	11.14 (45/404)	2.01	1.24 to 3.27	0.005	1.77	1.10 to 2.87	0.019
	35 to 44	8.05 (33/410)	1.46	0.87 to 2.44	0.153	1.31	0.78 to 2.18	0.302
	>45	5.53 (23/416)	1.00	‐	‐	1.00	‐	‐
Sex	Male	10.6 (37/349)	1.24	0.86 to 1.78	0.258	1.27	0.88 to 1.82	0.196
	Female	8.58 (84/979)	1.00	‐	‐	1.00	‐	‐
b)								
Age category	18 to 24	25.71 (18/70)	3.71	2.04 to 6.75	<0.001	2.47	1.38 to 4.44	0.002
	25 to 34	13.64 (42/308)	1.97	1.16 to 3.34	0.012	1.49	0.89 to 2.49	0.131
	35 to 44	9.49 (28/295)	1.37	0.78 to 2.42	0.276	1.07	0.61 to 1.86	0.817
	>45	6.92 (18/260)	1.00	‐	‐	1.00	‐	‐
Sex	Male	13.01 (32/246)	1.21	0.82 to 1.78	0.341	1.18	0.82 to 1.70	0.381
	Female	10.77 (74/687)	1.00	‐	‐	1.00	‐	‐
History of ART prior to testing campaign	No	22.12 (50/226)	2.79	1.97 to 3.97	<0.001	1.44	1.01 to 2.06	0.045
	Yes	7.92 (56/707)	1.00	‐	‐	1.00	‐	‐
History of LTFU prior to testing campaign	Yes	21.43 (84/392)	5.27	3.36 to 8.27	<0.001	4.24	2.64 to 6.81	<0.001
	No	4.07 (22/541)	1.00	‐	‐	1.00	‐	‐

PR, Prevalence Ratio; APR, Adjusted Prevalence Ratio; ART, Anti‐retroviral Therapy.

We then assessed the proportion of non‐disclosures solely among those 933 (70.3%) patients with a hospital identification ePTS number and with non‐missing data for covariates included in the model (Table 3b). Again, in this population, younger age was associated with non‐disclosure (Table 3b). Individuals aged 18 to 24 years, had a 2.47 fold greater risk of non‐disclosure (95% CI 1.38 to 4.44) compared to the reference category group. Those individuals with a history of LTFU prior to the time of the study had a 4.24 fold greater risk of non‐disclosure (95% CI 2.64 to 6.81) than those without prior recorded LTFU. Furthermore, individuals with no prior history of ART had a 1.44 fold greater risk of non‐disclosure (95% CI 1.01 to 2.06) than individuals enrolled in care but on ART (Table 3b). Sex was not significantly associated with non‐disclosure of HIV serostatus.

### 3.4 Impact of non‐disclosure on estimates of the first 90

**Figure 2:**
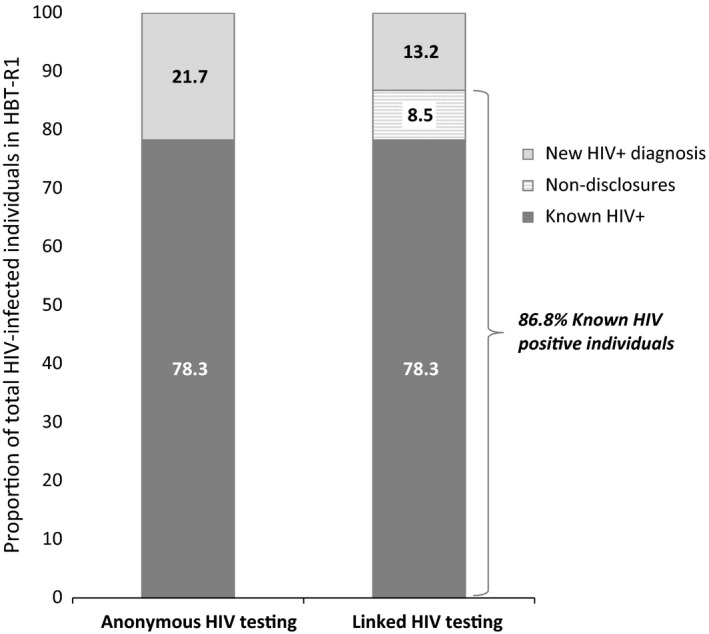
Impact of linking HIV testing results to patient registries on proportion of HIV‐infected individuals that know their status. (n = 497)

We then assessed non‐disclosure in the context of our population of PLWHIV in HBT‐R1 (N = 497) which included those previously identified by the record‐linkage algorithm (n = 67), self‐reported HIV‐status (n = 322), non‐disclosures (n = 42) and newly HIV‐diagnosed individuals (n = 66). In the absence of a mechanism to identify non‐disclosures, the apparent proportion of the 497 HIV‐positive individuals considered new HIV‐diagnoses would have been 21.7% (108/497) (95% CI 18.2 to 25.6) and the proportion who were aware of their status 78.3% (389/497) (95% CI 74.4 to 81.8). However, identification of the non‐disclosure population among those who tested positive modified these proportions. Figure 2 shows that shifting the 42/497 (8.5%, 95% CI 6.2 to 11.3) of non‐disclosures from the category of new HIV‐diagnoses to the category of *known HIV‐positive* reduces the proportion of new diagnoses to 13.2% (95% CI 10.4 to 16.5) and increases the proportion who are aware of their status to 86.8% (95% CI 83.4 to 89.6) (Figure 2).

## 4 Discussion

Our findings show that in a semi‐rural area of southern Mozambique, the proportion of individuals who did not disclose previous HIV‐positive status to a counsellor at the time of HIV‐testing varied according to testing venue and age. Among those individuals testing HIV‐positive in the VCT 13% underwent testing without disclosing a previous HIV‐diagnosis. This proportion was two‐fold higher in PICT and three‐fold higher in HBT. In the absence of an efficient and non‐anonymous clinical record tracking system for HIV‐testers, the apparent proportion of the HIV‐positive population who were aware of their serostatus in the community was 78.3%. However, after record‐linkage of HIV‐testers with the ePTS system, the adjusted proportion of the known HIV‐positive population rose to 86.8%, coming close to the UNAIDS target of 90% of the HIV‐positive population with an HIV‐diagnosis. Non‐disclosures were more likely to be young, have dropped‐out of care and not have a history of ART.

In Sub‐Saharan Africa, between 20 and 55% of the PLWHIV has been described to be unaware of their HIV‐status 4, 5, 22. Nevertheless, our results suggest that this proportion may include individuals who are in fact aware of their HIV‐status but prefer not to disclose and to repeat the test. This may lead to overestimation of the yield of new HIV‐diagnoses and overall HIV prevalence. Depending on the methods and back calculations used to estimate the undiagnosed HIV population, repeat HIV‐testing might overestimate the true proportion of PLWHIV who are still unaware of their HIV‐status 23.

To ensure that 90% of PLWHIV initiate ART, treatment delivery must be scaled‐up. Expansion of ART must go beyond treating new HIV cases and patients in follow‐up without ART criteria, to reach *known HIV‐positive individuals* who have dropped‐out of care and are more likely to undergo repeat testing without disclosing known HIV‐positivity to the counsellor. Indeed finding and re‐engaging HIV‐positive patients that have been lost at any step of the care cascade and may not disclose a prior HIV‐diagnosis is equally important to ensuring high yields of new HIV‐diagnoses. Several studies have shown a strong association between disclosure, social support and adherence 24, 25, 26, 27. PLWHIV who experienced positive social support were more likely to disclose their HIV‐status and link and retain in care, while those who perceived stigma or feared disclosing, presented lower levels of access to care 5, 26, 28, 29, 30. Despite this, very few studies have documented the magnitude of non‐disclosure to health providers 31, 32 and to our knowledge, no studies have measured non‐disclosure to the lay counsellor at the moment of HIV‐testing. Non‐disclosures may not be an isolated phenomenon. In a clinical PICT and VCT cohort in South Africa 33, 10% of the participants were identified as non‐disclosure after re‐testing whereas in a community based trial of self‐testing in Malawi, 26% of residents who self‐tested were already on ART 7, 34, 35 The reasons for non‐disclosure at time of counselling may include not believing the result, feeling that some time on treatment has cured them or stigma among others. Furthermore studies will shed light on the similarities and differences in non‐disclosure to a health provider versus to a partner.

The magnitude of non‐disclosures can distort programmatic indicators. At health facility level, non‐disclosures could overestimate basic indicators, such as the number of new HIV‐diagnoses and the total number of people living with HIV as well as underestimate the proportion of new HIV‐diagnoses linked to care. In Mozambique and other sub‐Saharan settings, most programmatic estimates of HIV‐testing coverage and linkage are based on comparing crude numbers of anonymous HIV‐positive tests versus number of people enrolling in care in an unlinked manner 36, 37. Here, we document single repeat HIV‐testing in HIV‐positive individuals who did not disclose their status to the lay counsellor. However, as long as testing information remains anonymous, individuals may repeat an HIV test several times without disclosing prior diagnoses. Thus, a proportion individuals classified as new diagnoses linked to care will actually be individuals lost over the care cascade who should be re‐engaged instead of re‐tested.

Identifying repeat HIV‐testers who are non‐disclosers is complicated by the generalized anonymity of the HIV‐diagnosis in Mozambique 38. With the increasing benefits of ART and challenges in linking new HIV‐diagnoses to care, WHO has revised its surveillance testing guidelines to move away from anonymous testing and encourage confidential linked HIV testing 14. In our study, under informed consent, HIV‐testing was not anonymous and thus allowed tracking these patients. Another important health system related obstacle is the reliance on paper charts when patients enrol in care. Digital entry of the clinical information in the ePTS is secondary to the clinical visit, where the information is recorded directly on the paper chart. *Mckay* et al. described the phenomenon of “medical multiplicity” in Maputo health facilities where one individual may have multiple charts 39. In addition, each health facility has their own clinical ePTS database, which complicates tracking silent transfers and migrants from one health post to another. Mechanisms such as the confirmation of the identity of individuals testing HIV‐positive prior to enrolment or the removal of anonymity of the HIV‐diagnosis implemented in our study allowed us to reduce the problem of ‘medical multiplicity’.

In this study, we did not conduct qualitative analyses to understand the reasons for non‐disclosure to the counsellor or to ascertain non‐disclosure to family members. However, the need for understanding the main reasons for non‐disclosure has increased in recent years as a greater proportion of the population is tested for HIV. Identifying barriers could reduce the magnitude of the problem and potentially improve adherence to ART and care due to the close relationship between disclosure and adherence 5, 26, 28, 29, 30. The main reasons for non‐disclosure at various levels (partners, family, friends, healthcare providers or even employers) are fear of discrimination or rejection 40, stigma 27, 30, 41, 42, 43 or previous negative experiences disclosing to a confident 28. In Mozambique, results from a national stigma survey have shown that more than 50% of people interviewed had experienced some form of discrimination in the previous year and less than 40% knew that they had rights as HIV patients 27, 44. Only half of the population of the survey had disclosed their status to their sexual partner or family 44.

Interventions that reduce stigma and empower PLWHIV will improve individual and social context outcomes. These interventions should be aimed at increasing sensitization, retention in care and de‐stigmatization of HIV at both individual and public health level 27, 30.

The main limitation of this study is related to the method of identification of the non‐disclosure population based on the ePTS, which only records individuals with a clinical visit rather than all those diagnosed and is independent by health facility. Thus, we were not able to include individuals who knew their HIV‐status but had not reached the point of a clinical visit or individuals that were enrolled in other health facilities. In addition, for HBT‐all rounds, our estimate of non‐disclosure is conservative because individuals that were identified as *known HIV‐positive* through the record ‐linkage algorithm were not provided with the opportunity to disclose their HIV condition. This is likely to lead to underestimation of the prevalence of non‐disclosure. With quality systems that allow the identification of previously diagnosed individuals, we will have the capacity to accurately measure the proportion of people still unaware of their HIV condition, after implementing all the strategies proposed to reach the first 90 goal.

## 5 Conclusions

In high HIV burden settings such as Southern Mozambique, close to 40% of people who tested positive within different HCT strategies had a prior HIV‐positive diagnosis. Repeat HIV‐testing of previously diagnosed individuals may contribute to medical multiplicity, wasting of scarce resources and distortion of programmatic indicators. In the absence of an efficient and non‐anonymous tracking system for HIV‐testers, repeat testing of non‐disclosing PLWHIV leads to misclassification between linkage and re‐engagement in care and can potentially lead to an underestimation of the first UNAIDS 90 objective. Since HIV‐status disclosure is believed to affect overall physical and mental health including disease transmission and the quality of relationships, understanding disclosure behaviour can impact many aspects of wellbeing of PLWHIV.

These findings point to a need, not only to link new HIV‐diagnoses to care but also to address the gap of re‐engagement at every step of the cascade in order to avoid extensive repeat HIV‐testing. Youths have become a target group for intensification of HIV‐testing. However, attention must be given to strategies of re‐engagement in care without repeating HCT. Intensifying the importance of disclosure and re‐engagement during counselling as well as ensuring appropriate psychosocial support are necessary to encourage PLWHIV to disclose their status.

## Competing interests

We declare no competing interests.

## Authors’ Contributions

DN conceived the study; DN, LFS and ELV designed the study; LFS, ELV, OA, CHS, NH, AN, EK and PV acquired the data. LFS, DN, ELV and OA analysed the data. LFS and DN wrote the first draft. All authors reviewed the manuscript and approved it for submission.

## Supporting information


**Appendix S1**. Supplementary methodology and results.
