# Supplementary material for: Monitoring progress towards the first UNAIDS target: understanding the impact of people living with HIV who re‐test during HIV‐testing campaigns in rural Mozambique
Source: J Int AIDS Soc. 2018 Apr 13;21(4):e25095. doi: 10.1002/jia2.25095 (PMC5898226; doi:10.1002/jia2.25095)
Supplement: Supplementary file 1 — Appendix S1. Supplementary methodology and results. [file JIA2-21-e25095-s001.docx]

**SUPPLEMENTARY APPENDIX:** Supplementary methodology and results.

**STUDY METHODS**

***Study setting***

The district is served by the Manhiça District Hospital (MDH), and 11 peripheral health posts. According to the national recommendations for HIV testing and counselling, HIV testing should be performed every 3 months. Thus, participants who referred having an HIV negative test performed in the previous 3 months did not undergo a new test unless they specifically requested it. Patients not eligible for ART (CD4>350 cells/mm3 in 2015 and 500 cells/mm3 in 2016) receive 6-month clinical evaluations plus CD4 testing. Pregnant women and TB patients with HIV follow a specific model of integrated care at the pre-natal and TB clinics, respectively.

***Study procedures for the linkage cohort***

The linkage prospective cohort study consecutively enrolled patients with a new HIV diagnosis from two clinic-based testing venues (VCT and PICT) and HBT between May 2014-June 2015 in the MDH. Inclusion criteria included receiving a first HIV-positive result, being at least 18 years of age and residing in the MDH catchment area. Those patients who at the time of the test were either pregnant women, co-infected with TB or known HIV positive cases were excluded. For the purpose of this study, PICT was implemented at the MDH adult triage clinic. HBT was performed by trained counselors using a list of adults randomly selected among the residents of the HDSS area and who were visited at their homes, offered HCT and asked to participate in the study. All participants with a new positive test result were given a referral slip to present to the MDH. Record Linkage techniques were applied to match demographic (HDSS) and clinical information (EPTS). First a deterministic linkage based on full name (first, middle and last name), date of birth and gender. For names that couldn't match a non-deterministic technique was employed. We used Jaro-Winkler and Levenshtein string distance algorithms (as implemented on RecordLinkage R package) applied to the full name and tolerated up to 80% similarity; and exact match on gender and date of birth. The performance of these techniques had been documented in fairly similar settings (18)The clinic based testing venues typically had staff members who guided patients from the testing unit to the MDH reception whereas for community testing, the individual was referred to the hospital at their own convenience. The study procedures did not influence the linkage to care beyond the HCT and facility-based guidance to the reception. HCT was performed individually, unless the participant requested to be tested with other family members or friends.

***Outcome definitions and statistical considerations***

The primary outcome for the linkage cohort analysis was linkage to care, a binary variable defined as having a first CD4 count available within three months of diagnosis. For the purpose of this study and assuming a 50% linkage to care rate in VCT, a sample size of 408 HIV-positive individuals in each testing group was estimated to be necessary to detect a difference of 10% in linkage to care by PICT or HBT as compared to VCT with an 80% power.

**ADDITIONAL RESULTS**

Study profile and baseline characteristics

Among individuals visited for HBT-all rounds, 68.7% (8071/11746) of the participants were eligible; 15.0% (1207/8071) self-reported HIV-positive status and 24.7% were later identified as non-disclosures (121/ 490).

**Figure S1. Separate study profiles for enrolment of testing HIV positive individuals in a) VCT, b) PICT and C) the four rounds of the community testing campaigns (HBT).** Percentages are calculated over the previous step.

a)

**Non-disclosure**

**118 (26.3%)**

448 (49.2%)

**NO 461**

**(50.8%)**

**Eligible**

**Tested HIV positive**

**VCT**

909 (46.5%)

418 outside MDH catchment area (46.0%)

5 pregnant (0.5%)

38 Refusals & other (4.3%)

**Newly HIV diagnosed**

**330 (73.7%)**

b)

**Non-disclosure**

**308 (42.1%)**

731 (69.9%)

**NO 315**

**(30.1%)**

**Eligible**

**Tested HIV positive**

**PICT**

1046 (53.5%)

282 outside MDH catchment area (27.0%)

4 pregnant (0.4%)

29 Refusals & other (2.8%)

**Newly HIV diagnosed**

**423 (57.9%)**

c)

**NO 3675**

**(31.3%)**

**Newly HIV diagnosed**

**369 (75.3%)**

4747 HIV-negative & Indeterminate (90.6%)

**HBT ROUNDS 1-4**

**11746 Home visits performed**

**Eligible**

8071 (68.7%)

**1207 Self-reported HIV+ (15.0%)**

**Tested**

**Non-disclosure**

**121 (24.7%)**

5237 (64.9%)

**Tested HIV positive**

490 (9.3%)

**NO 1627**

**(20.2%)**

144 Deaths (1.2%)

1648 out of the MDH catchment area (14.0%)

889 Missing (7.6%)

121 Pregnancies (1.0%)

24 Others (0.2%)

**849 Known HIV positive (7.2%)**

1287 Refusal (15.9%)

284 Self-reported negative (3.5%)

56 other (0.7%)

*Abbreviations*: Home-Based Testing (HBT) and Manhiça District Hospital (MDH).
